# Lambeau frontal médian dans la reconstruction d’un carcinome basocellulaire de la joue: à propos d’un cas

**DOI:** 10.11604/pamj.2016.25.245.11138

**Published:** 2016-12-21

**Authors:** Karim Bourra, Amin Belmahi

**Affiliations:** 1 Service de Chirurgie Plastique, Hôpital Al Farabi Oujda, Maroc; 2 Service Chirurgie Plastique, Hôpital Avicenne Rabat, Clinique des Nations Unis Rabat, Maroc

**Keywords:** Carcinome basocellulaire, carcinome spinocellulaire, lambeau frontal médian, lambeau local, mélanome malin, Basal cell carcinoma, squamous cell carcinoma, median forehead flap, local flap, malignant melanoma

## Image en médecine

Patiente âgée de 70 ans, victime d’un carcinome basocellulaire de la jonction naso-jugale gauche, où le carcinome est à cheval sur une partie la joue gauche et sur une partie gauche du dorsum nasal. La tumeur est d’apparition lentement progressive évoluant depuis 1 an. Une notion d’exposition solaire massive depuis l’enfance sans protection est retrouvée dans les antécédents. Une biopsie réalisée a évoqué un “CBC superficiel” nécessitant une ablation totale et complète avec des marges de sécurité entre 3 mm et 10 mm pour éviter une récidive locale, qui est la hantise de ses tumeurs cutanées à malignité locale. La patiente a par la suite bénéficié d’une intervention chirurgicale qui a permis de faire l’exérèse complète du CBC et la reconstruction de la perte de substance avec un lambeau local, qui est le lambeau frontal paramédian, prélevé au dépens du muscle frontal au front et mis en place sur la perte de substance avec une cicatrisation dirigée de la zone donneuse frontal. Nos diagnostics différentiels principaux sont un carcinome spinocellulaire et un mélanome malin qui ont été écartés. Le CSC touche les muqueuses et l’examen anapath est suffisant pour le différencier ‘un basocellulaire. Le mélanome malin a une localisation préférentielle à la plante du pied et cliniquement différent avec un examen anapath qui tanche aussi en faveur d’un carcinome basocellulaire… Notre patiente fut guérie par l’ablation complète du CBC dont les marges d’exérèses sont revenues saines même en profondeur. Patiente suivie à 3 mois, 6 mois et à 1 ans sans détecter de récidive locale.

**Figure 1:**
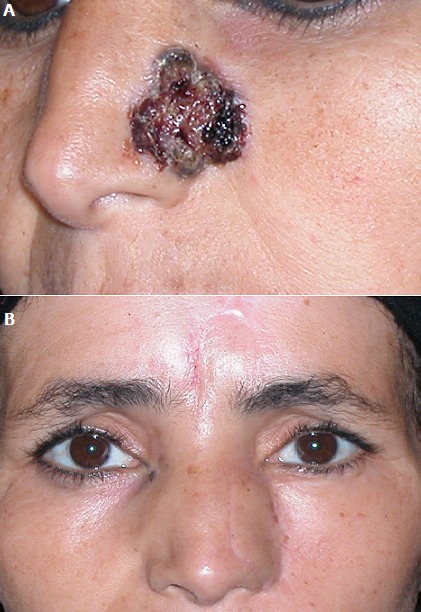
(A) carcinome basocellulaire jonction joue et nez gauche; (B) lambeau frontal médian mis en place sur la Pds (vue de Face). Zone frontale cicatrisée après cicatrisation dirigée

